# Proposing an Integrated Theory of Electronic Nicotine Delivery Systems Susceptibility Among Latinx and Non-Latinx Youth

**DOI:** 10.1007/s40615-025-02457-y

**Published:** 2025-05-06

**Authors:** Steven A. Branstetter, Joshua Muscat

**Affiliations:** 1 Department of Biobehavioral Health, The Pennsylvania State University, 219 BBH Building, University Park, PA 16802 USA; 2 Division of Epidemiology, Department of Public Health Sciences, The Pennsylvania State University College of Medicine, 700 HMC Crescent Road, Hershey, PA 17033 USA

**Keywords:** ENDS (Electronic Nicotine Delivery Systems), Latinx youth, Susceptibility, Parental influence, Behavioral theory

## Abstract

**Introduction:**

Despite a reduction in overall cigarette smoking rates in the last several decades, recent increases in the use of electronic nicotine delivery systems (ENDS), such as e-cigarettes and Juul devices, prompted the Surgeon General to declare the use of such products to be an “epidemic.” Along with the increase in the overall incidence of ENDS use, there are corresponding disparities in initiation, attitudes, and use patterns among Latinx populations.

The present study sought to examine how elements of several behavioral theories, including parent, peer, media and environmental factors, are associated with a comprehensive measure of ENDS use susceptibility among both Latinx and non- Latinx youth.

**Methods:**

Data from 9630 adolescents between the ages of 12 and 17 years were collected in wave 3 of the Population Assessment of Tobacco and Health (PATH). Data were used to create and test a covariance structural path model examining the effects of parental modeling, communication, attitudes, rule-setting, and peer perceptions on ENDS use susceptibility. Multigroup model comparisons between Latinx and non-Latinx youth were completed.

**Results:**

Despite non-Latinx youth having more risk factors associated with ENDS use susceptibility, including parental ENDS use, more lenient rules regarding ENDS use in the home, and less perceived harm from ENDS use, Latinx youth had higher overall susceptibility. Additionally, despite predictions from prior research and theory, perceived social norms regarding ENDS use were not associated with susceptibility among non-Latinx youth. Parental ENDS use and having ENDS products available in the home were associated with greater susceptibility in Latinx, but not non-Latinx youth.

**Conclusions:**

Key differences in ENDS susceptibility between Latinx and non-Latinx youth warrant both further investigation and increased culturally tailored prevention programming.

## Introduction

In 2019, there was a global call to action to address what U.S. Surgeon General Jerome Adams called an “epidemic” of the use of electronic nicotine delivery systems (also known as “vaping”) among youth in the USA [18]. Researchers have become concerned that the growth in the use of electronic nicotine delivery systems (ENDS) may represent a tipping point in nicotine dependence among already vulnerable populations [40]. While overall cigarette smoking rates were declining, the rates of smoking and smoking onset among Latinx populations (e.g., Latino or Hispanic) have continued to increase since 2000 [3, 11]. Similarly, fewer Latinx smokers utilize empirically supported treatment options during cessation attempts and are less likely to get advice to quit from health professionals [4, 12].

Likewise, there are disparities among Latinx individuals in the use of electronic nicotine delivery systems (ENDS, also known as electronic cigarettes or “vaping” devices), in terms of initiation, attitudes, and use patterns [2, 17, 21, 33, 41, 43]. Data from the National Youth Tobacco Survey suggest that among minority adolescents in the USA, the odds of ENDS initiation are highest among Latinx populations [16], and that Latinx youth have a greater curiosity about using ENDS products and are more likely to start using ENDS than non-Latinx peers [6, 25]. E-cigarettes are currently the most commonly used tobacco product among Latinx middle and high school students [29].

Different behavioral theories have been developed to explain the use of tobacco products among youth. The Theory of Planned Behavior and other behavioral theories (e.g., Behavioral Reasoning Theory [34]) emphasize the concept of intention as a key predictor of future behavior [1], including the use of ENDS [36]. Behavioral intent is one of several indicators of the broader concept of susceptibility, defined as a lack of commitment to avoid a specific behavior [24]. Susceptibility is a strong predictor of later cigarette, ENDS, and hookah use [7] and may predict smoking experimentation better than even parental or peer smoking behaviors [31]. Validated measures of susceptibility have traditionally used three items to assess the commitment to avoid smoking: (1 the intent to smoke “soon” (immediate intent, (2 whether one would smoke a cigarette if offered by a friend (direct peer influence, and (3 whether one intends to use cigarettes in 1 year (future intent [31],). More recently, the construct of curiosity has also emerged as a significant predictor of intent and progression to use cigarettes [28, 32], hookah [14], and even ENDS [13, 19, 25, 42]. Latinx youth, in particular, may be the most curious to use these products [13]. Adding curiosity to traditional measures of susceptibility can significantly improve the prediction of adolescents who may progress to experimentation and use of tobacco products [28].

Behavioral theories such as the Health Beliefs Model (HBM), the Theory of Planned Behavior (TPB), the Social-Ecological Model (SEM), and the Social Cognitive Theory (SCT) include empirically supported factors associated with the intent to use ENDS. For example, SCT suggests that parental use of ENDS would increase a youth’s intent to use by providing a role model for use [5]. Likewise, the TPB suggests that perceptions of ENDS use being an approved social behavior would increase intent to use ENDS because of a positive perceived social norm [16, 30]. The HBM would predict that intent to use ENDS would be low if youth perceived ENDS use as harmful. The SEM would suggest that environmental contexts, such as those provided by parents—including advice not to use and expressed negative perceptions of ENDS—would impact adolescents’ intent to use. By integrating these models, we could account for the multifaceted nature of ENDS use, capturing both cognitive and social-environmental influences. This approach acknowledges the existing research that susceptibility to substance use arises not only from individual risk perceptions and intentions but also from external social and structural factors. Indeed, it has been suggested that combining key factors from multiple theoretical perspectives may be the most effective method in predicting future use of these products [8]. In our models, the role of observational learning, peer influence, and outcome expectancies in shaping youth behavior reflects SCT,the role of attitudes, subjective norms, and perceived behavioral control reflects key elements of TPB; and risk perception, perceived susceptibility, and perceived benefits and barriers highlight features of the HBM. Finally, SEM allows for a broader contextualization by recognizing the interplay of individual, interpersonal, community, and societal factors that contribute to susceptibility. By proposing this integrated theoretical model of susceptibility to ENDS, we may provide a more holistic ability to identify factors associated with youth ENDS use onset and help best inform prevention and intervention programming.

## Methods

### Data Source

Data for the present study were collected as part of the Population Assessment for Tobacco and Health (PATH) study, a nationally representative, longitudinal cohort study including nearly 50,000 participants over the age of 12. Details regarding sampling and data collection are detailed and available on the National Addiction & HIV Data Archive Program website [27]. The current study utilized data from Wave 3, collected between October 2015 and October 2016, and included data from both adult parents and their minor children. The IRB at Pennsylvania State University granted exempt status to the present study due to the deidentified nature of the publicly available data.

### Measures

#### Prior Use (Youth ENDS Use)

Youth were assessed at Wave 3 regarding their use of any ENDS device with the yes/no item, “*Have you ever used an electronic nicotine product, even one or two times*?” To explore susceptibility to use ENDS, youth who had used ENDS at or before Wave 3 were excluded from the present study.

#### Curiosity (ENDS Curious)

Youth participants were asked, “*Have you ever been curious about using an electronic nicotine product? (Electronic nicotine products include e-cigarettes, vape pens, personal vaporizers and mods, e-cigars, e-pipes, e-hookahs and hookah pens.)*” Responses were recorded using a 4-point Likert scale where 1 = not at all curious, 2 = a little curious, 3 = somewhat curious, and 4 = very curious.

#### Intent

Youths were asked, “*Do you think you will try an electronic nicotine product in the next year?*” (ENDS 1 year). They were also asked, “*Do you think you will try an electronic nicotine product soon?*” (ENDS soon), and “*Would you use an electronic nicotine product if one of your best friends offered you one*?” (ENDS offered). Each of these items used a 4-point Likert scale, where 1 = definitely yes, 2 = probably yes, 3 = probably not, and 4 = definitely not.

#### Perceived Norms (ENDS Norms)

Youth participants were asked, “*In general, do you think most people disapprove of using e-cigarettes or other electronic nicotine products*?” Responses were recorded using a 4-point Likert scale where 1 = definitely yes, 2 = probably yes, 3 = probably not, and 4 = definitely not.

#### Perceptions of Peer Use

Youths were asked, “*How many of your best friends use e-cigarettes or other electronic nicotine products*?” (ENDS peer use), using a 5-point Likert scale where 1 = none, 2 = a few, 3 = some, 4 = most, and 5 = all. Youths were also asked, “*Thinking about the people who are important to you, how would you describe their views on using e-cigarettes or other electronic nicotine products*?” (ENDS peer views). This item used a 5-point Likert scale where 1 = very positive, 2 = somewhat positive, 3 = neither positive nor negative, 4 = somewhat negative, and 5 = very negative.

#### Parental Use (ENDS Use, Parent)

Parent ENDS use was assessed with the yes/no item “*Do you now use electronic nicotine products*?”.

#### Parental Discussion (ENDS Parent Talks)

Youths were asked the yes/no question, “*In the past 12 months, have your parents or guardians talked with you, even once, about not using e-cigarettes or other electronic nicotine products*?”.

#### Household Restrictions (ENDS Home Rules)

Parents were asked, “*Which statement best describes the rules about using [e-cigarettes and other electronic nicotine products] inside your home*?” with response options of 1 = It is not allowed anywhere or at any time inside my home, 2 = It is allowed in some places or at some times inside my home, or 3 = It is allowed anywhere and at any time inside my home.

#### Availability in Home

Parents were asked the yes/no question: “*Do you think any tobacco products or electronic nicotine products (such as e-cigarettes) might be available to your child at your home*?” (ENDS available). Additionally, youths were asked the yes/no question: “*Not including yourself, does anyone living in your home own an e-cigarette or other electronic nicotine product*?” (ENDS owned).

#### Harm Perceptions (ENDS Harm, Youth; ENDS Harm, Parents)

Both youth and parents were asked about their perception of the general harmfulness of ENDS with the item: “*How much do you think people harm themselves when they use e-cigarettes or other electronic nicotine products*?” Responses were recorded on a 4-point Likert scale, where 1 = No harm, 2 = Little harm, 3 = Some harm, and 4 = A lot of harm.

#### Access Difficulty (ENDS Ease)

Youth participants were asked, “*How easy do you think it is for people your age to buy e-cigarettes or other electronic nicotine products in a store*?” Responses were recorded using a 5-point Likert scale, where 1 = Very easy, 2 = Easy, 3 = Neither easy nor difficult, 4 = Difficult, and 5 = Very difficult.

#### Social Media Exposure (ENDS Social Media)

Youths were asked the yes/no question, *“In the past 30 days, have you noticed e-cigarettes or other electronic nicotine products being advertised [on websites or social media sites]?”.*

### Composite Variable

#### Susceptibility Index (ENDS Susceptibility)

An average “susceptibility” variable was created that included (1) ENDS intent, including ENDS soon, ENDS 1 year, and ENDS offered and (2) ENDS curious.

### Analytic Approach

**Fig. 1:**
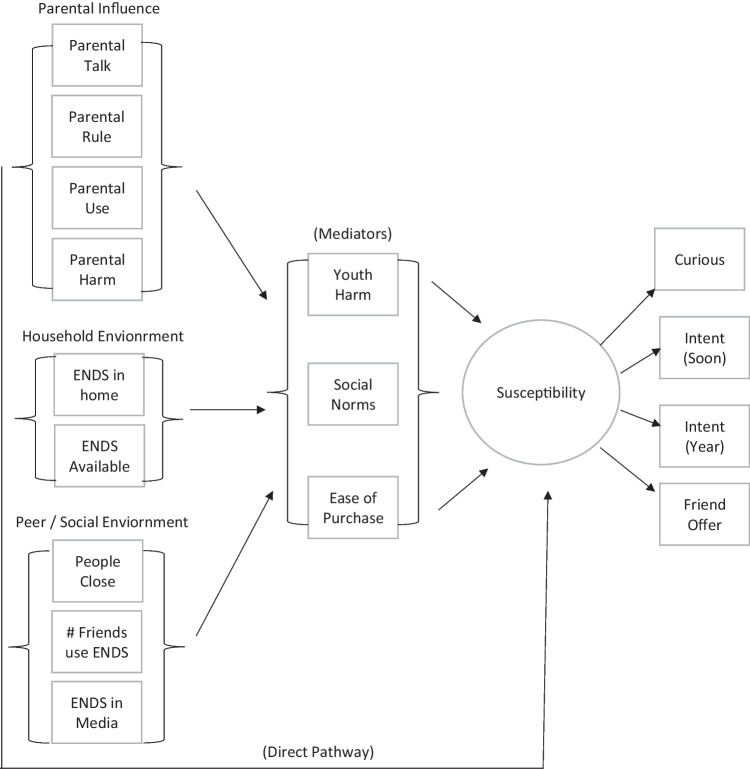
Conceptual model for ENDS susceptibility

Descriptive and independent sample *t*-tests and chi-square were completed using SPSS (version 28). A latent confirmatory factor analysis was conducted on the ENDS susceptibility construct using AMOS software (version 7). Next, multi-group path analyses are conducted to examine the relation between ENDS use, parent, ENDS parent talk, and ENDS home rules on ENDS curious, ENDS harm, youth and ENDS intent; see conceptual model in Fig. 1. This approach allowed for the determination of both direct and indirect effects, as well as overall effects of variables on one another, and to examine differences between Latinx and non-Latinx youth. In this analysis, 1000 bootstrap samples were generated to obtain more reliable estimates of path coefficients, standard errors, and confidence intervals.

### Sample

A total of 9630 individuals including youth between the ages of 12 and 17 years of age who had never used ENDS products at Wave 3, and one of their parents, were included in the present study. The sample was 51.4% (*n* = 4943) male and 48.6% female (*n* = 4666), with a total of 21 participants who were missing data on this variable due to “don’t know” or “refused to answer” responses. A total of 2759 participants identified as Hispanic with 6542 identifying as non-Hispanic. A total of 329 participants were missing data on race due to a “don’t know” or “refused” response. Data were checked for large amounts of missingness; however, no single variable had more than 5% missing, a threshold below which methods such as data imputation have minimal utility [35]. As such, analyses used listwise deletion (e.g., *t*-test and *X*^2^) or full maximum likelihood estimation to handle missing data in analyses.

## Results

Table 1 compares all the variables between Latinx and non-Latinx youth. Key findings include that parents of Latinx youth, compared to parents of non-Latinx youth, were nearly twice as likely to have used ENDS products in the previous 30 days (ENDS use, parent; 4.1% vs. 2.2%; *X*^2^ (1,9113) = 21.25, *p* < 0.001) and were more than twice as likely to allow ENDS use anywhere within the home (ENDS home rules; 8.2% vs. 3.8%, *t*(7063) = − 13.34, *p* < 0.001). In contrast, non-Latinx youth were almost twice as likely to have someone in the home who owns an ENDS device (ENDS owned; 10.9% vs. 5.8%; *X*^2^ (1,8805) = 56.38, *p* < 0.001) and twice as likely to have tobacco or ENDS products available within their households (ENDS available; 13.2% vs. 6.5%; *X*^2^ (1,9295) = 87.88, *p* < 0.001. Using the ENDS susceptibility variable, Latinx youth were slightly (*M* = 3.80, *SD* = 0.38) but significantly more susceptible to ENDS use than non-Latinx youth (*M* = 3.82, *SD* = 0.35), *t*(4904) = − 2.57, *p* = 0.005.

**Table 1:** Descriptive statistics/frequencies

	Latinx (N = 2159)		Non-Latinx		Results	*p*
	*N*	%	*N*	%		
ENDS use, parent					*X*^2^ (1,9113)=21.25	<0.001
1. Yes	59	2.2	265	4.1		
2. No	2648	97.8	6141	95.9		
ENDS home rules					*t*(7063) = − 13.34	<0.001
1. Not allowed in home	2484	91.7	5183	81.2		
2. Allowed some places/times in home	123	4.5	671	10.5		
3. Allowed anywhere/any time in home	103	3.8	526	8.2		
Ends parent talk					*X*^2^ (1,9177)=29.06	<0.001
1. Yes	1185	43.4	2407	37.4		
2. No	1548	56.6	4037	62.6		
ENDS harm, parent					*t*(5870) = 17.36	< 0.001
1. No harm	25	.9	91	1.5		
2. Little harm	120	4.5	561	9.0		
3. Some harm	589	22.1	2315	37.2		
4. A lot of harm	1936	72.5	3257	52.3		
ENDS harm, youth					*t*(5314)=4.61	<0.001
1. No harm	40	1.5	89	1.4		
2. Little harm	184	6.7	571	8.8		
3. Some harm	760	27.8	2024	31.2		
4. A lot of harm	1747	64.0	3793	58.6		
ENDS norms					*t*(9026)=1.65	.05
1. Definitely yes	377	14.1	924	14.5		
2. Probably yes	1208	45.2	2914	45.9		
3. Probably not	760	27.8	2081	32.8		
4. Definitely not	1747	64	3793	58.6		
ENDS curious					*t*(8838)=-.67	.25
1. Very curious	35	1.4	84	1.3		
2. Somewhat curious	123	4.8	263	4.2		
3. A little curious	382	14.8	947	15.1		
4. Not at all curious	2034	79.0	4972	79.3		
ENDS soon					*t(*4432)=−3.40	<0.001
1. Definitely yes	3	.1	8	.1		
2. Probably yes	24	.9	41	.7		
3. Probably not	359	13.9	710	11.3		
4. Definitely not	2193	85	5511	87.9		
ENDS One Year					*t*(4558)=−3.26	<0.001
1. Definitely yes	3	.1	8	.1		
2. Probably yes	37	1.4	90	1.4		
3. Probably not	414	16.1	800	12.8		
4. Definitely not	2123	82.4	5376	85.7		
ENDS owned					*X*^2^ (1,8805)=56.38	<0.001
1. Yes	148	5.8	681	10.9		
2. No	2417	84.2	5559	89.1		
ENDS available					*X*^2^ (1,9295)=87.88	<0.001
1. Yes	178	6.5	861	13.2		
2. No	2578	94.2	5678	86.8		
ENDS Social Media					*X*^2^ (1,9127)=11.81	<0.001
1. Yes	506`	18.7	1408	21.9		
2. No	2198	93.5	5015	78.1		
ENDS peer use					*t*(9239)=.42	.68
1. None	2313	84.3	5507	84.8		
2. A few	321	11.7	746	11.5		
3. Some	89	3.2	176	2.7		
4. Most	15	.5	46	.7		
5. All	7	.3	21	.3		
ENDS peer view					*t*(5413)=2.72	0.007
1. Very positive	58	2.1	175	2.7		
2. Positive	102	3.8	342	5.3		
3. Neither positive or negative	341	12.6	845	13.1		
4. Negative	582	21.5	1280	19.9		
5. Very negative	1619	59.9	3797	59		
ENDS ease					*t*(9027)=−1.33	.18
1. Very easy	336	12.6	730	11.5		
2. Somewhat easy	729	27.2	1744	27.5		
3. Somewhat difficult	806	30.1	1889	29.7		
4. Very Difficult	806	30.1	1989	31.3		
ENDS offered					*t*(4695)=−1.90	.06
5. Definitely yes	6	.2	23	.4		
6. Probably yes	82	3.2	185	2.9		
7. Probably not	420	16.3	890	14.2		
8. Definitely not	2061	80.2	5174	82.5		

An initial confirmatory factor analysis (*CFA*) was conducted to demonstrate that the latent ENDS susceptibility factor had an adequate model fit. The susceptibility factor had four indicators: (1) ENDS curious, (2) ENDS intent soon, (3) ENDS intent 1 year, and (4) ENDS offered. The susceptibility factor demonstrated acceptable model fit, *X*^2^ = 263.20 (*df* = 2, *p* < 0.001), *RMSEA* = 0.06, *NFI* = 0.97, and a *CFI* = 0.98. All items loaded onto the latent susceptibility factor at acceptable levels: ENDS curious *β* = 0.70, *p* = < 0.001, ENDS soon *β* = 0.86, *p* = < 0.001, ENDS 1 year *β* = 0.90, *p* = < 0.001, and ENDS offered *β* = 0.81, *p* = < 0.001. See Fig. 1 for the conceptual model of factors loading on ENDS susceptibility construct.

The overall model tested, represented as a conceptual model in Fig. 1, demonstrates good model fit: *X*^2^ = 628.35 (*df* = 82, *p* < 0.001), *RMSEA* = 0.02, *NFI* = 0.98, and a *CFI* = 0.98; the results of the full model are presented in Table 2. Key findings suggest that the mediator variables of ENDS harm, youth and ENDS ease were significantly associated with ENDS susceptibility for both Latinx and non-Latinx youth. However, ENDS norms were associated with ENDS susceptibility among Latinx youth only.

**Table 2:** Results of path models: factors on mediators and susceptibility

	Ease of ENDS purchase		Perceived harm		Social norms		Susceptibility	
	Latinx	Non-Latinx	Latinx	Non-Latinx	Latinx	Non-Latinx	Latinx	Non-Latinx
ENDS rule	.04	.05**	−.03	−.05**	.04**	0.00	.04	−.01
ENDS harm, parent	−.01	−.03*	.12***	−.05*	−.01	.13***	−.01	.01
ENDS use, parent	−.02	.01	−.02	.02	−.01	.01	−.05*	−.02
ENDS parent talk	−.03	0.00	−.06**	− 04***	.03**	−.04**	−.01	0.00
ENDS available	0.001	−.01	.02	−.01	.02	.02	−.01	.01
ENDS owned	−.03	−.03	.04	−.02	.01	.01	.06**	.01
ENDS peer use	−.13***	−.15***	−.10***	.08***	.10***	−.12***	−.21***	−.24***
ENDS peer view	.04	.03*	.15***	−.13***	−.20***	.20***	.10***	.11***
ENDS social media	.13***	.11***	.03***	−.02***	−.04**	.05***	.06**	.07***

^*^ *p* <.05; ** *p* <.01; *** *p* < 0.001; values reflect unstandardized beta coefficients

### Associations with Mediator Variables

#### ENDS Ease of Purchase

For Latinx and non-Latinx youth, ENDS peer use and ENDS social media were associated with the perception that purchasing ENDS is easier for youth (ENDS ease). For non-Latinx youth, the perception that people close to them holding negative views of ENDS use (ENDS norms) was associated with perceptions that purchasing ENDS is more difficult for youth (ENDS ease). Interestingly, among non-Latinx youth, stricter ENDS rules and greater parental perceptions of ENDS harm were both associated with a higher perceived ease of purchasing ENDS.

#### Youth Perceptions of Harm (ENDS Harm, Youth)

Higher parental perceptions of harm (ENDS harm, parent), having had a parent talk about ENDS use (ENDS parent talk), fewer friends who use ENDS (ENDS peer use), and having people who are close with negative views of ENDS (ENDS peer views) were all associated with increased perceptions of harm from ENDS among both Latinx and non-Latinx youth. Exposure to ENDS advertisements (ENDS social media) was associated with perceptions ENDS are less harmful among non-Latinx youth only.

#### Perceptions of Social Norms (ENDS Norms)

Among both Latinx and non-Latinx youth, fewer friends who use ENDS (ENDS peer use), perceptions that people close have negative views of ENDS (ENDS peer views), and having had a parental talk about ENDS (ENDS parent talk) were all associated with increased perceptions that most people disapprove of ENDS (ENDS norms). For Latinx youth, increased parental perceptions of harm (ENDS harm, parent) were also associated with perceptions of ENDS norms. For non-Latinx youth only, ENDS social media was associated with perceptions that fewer people disapprove of ENDS. Parental household rules (ENDS home rules) had the opposite effect on perceptions of social norms between Latinx and non-Latinx youth. For Latinx youth, more restrictive household rules were associated with a lower likelihood of believing that most people disapprove of ENDS, whereas for non-Latinx youth, restrictive household rules were associated with an increased perception of ENDS disapproval. See Table 2 for full results.

#### Mediator Associations with ENDS Susceptibility

Perceptions that ENDS are harmful (ENDS harm, youth) and that ENDS are more difficult to purchase for youth (ENDS ease) are associated with lower ENDS susceptibility among both Latinx and non-Latinx youth. Among Latinx youth, perceptions that most people disapprove of ENDS (ENDS norms) were also associated with lower ENDS susceptibility. However, perceptions of social norms were not associated with ENDS susceptibility among non-Latinx youth. See Table 3 for results.

**Table 3:** Results of path model: mediators on susceptibility

	Susceptibility	
	Latinx	Non-Latinx
Ease of ENDS purchase	.04*	.05***
Perceived harm	.22***	.22***
Social norm	−.04*	−.02

^*^ *p* <.05; ** *p* <.01; *** *p* < 0.001; values reflect unstandardized beta coefficients

#### Direct Associations with ENDS Susceptibility

For both Latinx and non-Latinx youth, the more friends who use ENDS (ENDS peer use), greater exposure to ENDS in the media (ENDS social media), perceptions that people who are close view ENDS more positively (ENDS peer views) were all associated directly with higher ENDS susceptibility. Additionally, ENDS parent use and having someone else in the home who owns an ENDS device (ENDS owned) was associated with higher ENDS susceptibility among Latinx youth only.

## Discussion

This present study provides insights into the factors influencing ENDS susceptibility among Latinx and non-Latinx youth, revealing distinct cultural and familial influences on ENDS use and perceptions. The findings highlight the importance of considering cultural contexts, such as parental rules and social norms, when developing prevention and intervention strategies for youth tobacco and ENDS use. Additionally, the study underscores the significant role of peer and media influences, as well as parental attitudes and household regulations, in shaping youth behavior. These results are crucial for informing targeted, culturally relevant public health campaigns and policies aimed at reducing youth ENDS use across diverse communities. The results also highlight the utility of the proposed integrated theory in understanding the contributions of individual perceptions, peer and parental environmental factors, and other external factors, such as social media, may play in the susceptibility to ENDS onset.

Overall, the results reflect that peers are among the greatest proximal influences on adolescent behavior and decision-making in general [37, 38], and social media has a direct association with increased ENDS initiation and use [22]. Conversely, in both Latinx and non-Latinx youth, parental influence was largely limited to increasing perceptions of harm and reducing social norms via a specific talk about ENDS. Other unmeasured parental behaviors may have differential influences on ENDS susceptibility among Latinx and non-Latinx youth, suggesting the need for additional research.

Consistent with several behavioral theories, youth perceptions of harm and perceptions of easy access to ENDS were associated with increased susceptibility to ENDS use. However, perceptions of social norms of ENDS use were associated with ENDS susceptibility among Latinx youth only. Perceptions of general disapproval of smoking have been inconsistently associated with smoking initiation among adolescents [10]. The present study’s finding that perceptions of general disapproval of ENDS reduces susceptibility among Latinx, but not among non-Latinx, youth is noteworthy. This difference may be attributed to Latinx cultural values including *familismo*, *respeto*, and *bien educado*, which emphasize the importance of respect for others and social customs [23]. As such, the view that others within the community may disapprove of ENDS use may have greater influence among Latinx youth.

For many behavioral theories, including Social Cognitive Theory and the Theory of Planned Behavior, a parental role model has a significant influence on youth perceptions and behavior. Parental smoking is among the strongest factors associated with youth smoking uptake [39], and parental use of ENDS is associated with youth ENDS use [15]. However, having a parent who uses ENDS did not have a direct association with youth ENDS susceptibility among non-Latinx youth. Although parents of non-Latinx youth did influence key variables, these influences were not always what might have been predicted by theory. For example, non-Latinx youth with more restrictive parental rules regarding household use of ENDS tended to perceive that it would be easier to purchase ENDS (i.e., fewer barriers to ENDS use). Likewise, non-Latinx youth with parents who viewed ENDS as more harmful tended to view purchasing ENDS as easier. Perceptions of ease of access to tobacco products has typically been associated with peer and parental use, positive perceived norms and attitudes [20], but other parenting activities have also been found to influence perceptions of access to tobacco products. For example, even lax parental rules regarding watching restricted movies have been associated with an increased perception of tobacco accessibility [9]. Again, these results may reflect culture whereas parents of Latinx youth may have more of an influence on alcohol and marijuana use onset than do parents of non-Latinx youth [26].

Finally, it is noteworthy that despite non-Latinx youth having relatively higher risk factors for ENDS use in many domains assessed used in the present study, Latinx youth were modestly, yet significantly, more susceptible to ENDS use than non-Latinx youth. Specifically, non-Latinx youth had parents who used ENDS more frequently, perceived ENDS as less harmful, and maintained more permissive household rules. They also had greater access to ENDS products at home, were less likely to have discussed ENDS use with their parents, experienced more exposure to ENDS on social media, and personally viewed ENDS as less harmful. The fact that Latinx youth were nevertheless modestly higher on an average measure of susceptibility is of some concern. Future studies should explore how specific cultural factors shape perceptions of parental and peer behaviors, influence social media exposure, and contribute to unique cultural, familial, or community dimensions that may increase susceptibility to ENDS and other tobacco use among Latinx youth. Indeed, there is a great deal of heterogeneity among Latinx populations, and future research should strongly consider how variations in acculturation, socioeconomic background, or generational differences might impact these findings.

Limitations in the present study include the use of cross-sectional data, which limit making causal inferences. Instead, we have developed plausibility models based on prior research and behavioral theory that allowed for the examination of associations between factors. We use the term “direct effect” to reflect a direct relationship between variables, and the term “indirect effect” when a relation between variables operates through a mediating variable. These terms are not intended to imply causation. Whereas this study has identified potential areas of disparities in susceptibility to ENDS use among Latinx youth, it is unable to address potential cultural or other mechanisms for these disparities. Additionally, the reliance on self-reported surveys may introduce biases, including social desirability bias. Further, non-response bias could impact findings if youth or parents who chose not to participate differ systematically from those who did. Future research should consider alternative data collection methods, such as longitudinal designs or objective measures, to mitigate these limitations.

Overall, the present study did highlight key areas that may be helpful in furthering tailor prevention programs for Latinx youth, such as the role of parental ENDS use and the availability of ENDS products in the home and perceptions of social norms.

## Data Availability

The data used in this study are publicly available from the Population Assessment of Tobacco and Health (PATH) Study, which is conducted by the National Institute on Drug Abuse (NIDA) and the Food and Drug Administration (FDA). Public-use files can be accessed through the National Addiction & HIV Data Archive Program (NAHDAP) at https://www.icpsr.umich.edu/icpsrweb/NAHDAP/studies/36498. Restricted-use files are available upon application and approval for researchers meeting eligibility criteria.
