# Correction to: Proposing an Integrated Theory of Electronic Nicotine Delivery Systems Susceptibility among Latinx and Non-Latinx Youth

**DOI:** 10.1007/s40615-025-02822-x

**Published:** 2025-12-24

**Authors:** Steven A. Branstetter, Joshua Muscat

**Affiliations:** 1 Department of Biobehavioral Health, The Pennsylvania State University, 219 BBH Building, University Park, PA 16802 USA; 2 Division of Epidemiology, Department of Public Health Sciences, The Pennsylvania State University College of Medicine, 700 HMC Crescent Road, Hershey, PA 17033 USA

**Correction to: Journal of Racial and Ethnic Health Disparities**

10.1007/s40615-025-02457-y.

The surname of coauthor Joshua Muscat was misspelled in the article as originally published.The original article has been corrected.

